# Recognizing Cross-Institutional Fiscal and Administrative Barriers and Facilitators to Conducting Community-Engaged Clinical and Translational Research

**DOI:** 10.1097/ACM.0000000000003893

**Published:** 2021-03-30

**Authors:** Lori Carter-Edwards, Mary E. Grewe, Alecia M. Fair, Carolyn Jenkins, Natasha J. Ray, Alicia Bilheimer, Gaurav Dave, Marcella Nunez-Smith, Alan Richmond, Consuelo H. Wilkins

**Affiliations:** 1 **L. Carter-Edwards** is associate professor, Public Health Leadership Program, adjunct faculty in epidemiology and health behavior, Gillings School of Global Public Health, and director, Community and Stakeholder Engagement (CaSE) Program, North Carolina Translational and Clinical Sciences Institute (NC TraCS), University of North Carolina at Chapel Hill, Chapel Hill, North Carolina; ORCID: https://orcid.org/0000-0002-5552-136X.; 2 **M.E. Grewe** is project manager/qualitative research specialist, CaSE Program, NC TraCS, University of North Carolina at Chapel Hill, Chapel Hill, North Carolina; ORCID: https://orcid.org/0000-0002-9979-4394.; 3 **A.M. Fair** is research assistant professor of medicine, Division of Geriatric Medicine, Vanderbilt University Medical Center, Nashville, Tennessee; ORCID: https://orcid.org/0000-0003-0144-1425.; 4 **C. Jenkins** is professor and Ann Darlington Edwards Endowed Chair, College of Nursing, and community engagement codirector, South Carolina Clinical & Translational Research Institute, Medical University of South Carolina, Charleston, South Carolina; ORCID: https://orcid.org/0000-0001-5506-7657.; 5 **N.J. Ray** is core services manager, New Haven Healthy Start, The Community Foundation for Greater New Haven, New Haven, Connecticut.; 6 **A. Bilheimer** is administrative director, CaSE Program, NC TraCS, University of North Carolina at Chapel Hill, Chapel Hill, North Carolina.; 7 **G. Dave** is associate professor of medicine (social medicine), School of Medicine, and associate director, Center for Health Equity Research, University of North Carolina at Chapel Hill, Chapel Hill, North Carolina; ORCID: https://orcid.org/0000-0003-0825-1595.; 8 **M. Nunez-Smith** is associate professor of medicine (general medicine) and epidemiology (chronic diseases), associate dean, Health Equity Research, director, Equity Research and Innovation Center, director, Center for Research Engagement, core faculty, National Clinician Scholars Program, deputy director of health equity research and workforce development, Yale Center for Clinical Investigation, and director, Yale-Commonwealth Fund Fellowship in Health Equity Leadership, Yale University, New Haven, Connecticut; ORCID: https://orcid.org/0000-0003-2797-4756.; 9 **A. Richmond** is executive director, Community-Campus Partnerships for Health, Raleigh, North Carolina.; 10 **C.H. Wilkins** is professor of medicine, Division of Geriatric Medicine, and vice president of health equity and associate dean for health equity, Vanderbilt University Medical Center, Nashville, Tennessee; ORCID: https://orcid.org/0000-0002-8043-513X.

## Abstract

This qualitative study examined fiscal and administrative (i.e., pre- and post-award grants process) barriers and facilitators to community-engaged research among stakeholders across 4 Clinical and Translational Science Awards (CTSA) institutions.

**Method:**

A purposive sample of 24 key informants from 3 stakeholder groups—community partners, academic researchers, and research administrators—from the CTSA institutions at the University of North Carolina at Chapel Hill, Medical University of South Carolina, Vanderbilt University Medical Center, and Yale University participated. Semistructured interviews were conducted in March–July 2018, including questions about perceived challenges and best practices in fiscal and administrative processes in community-engaged research. Transcribed interviews were independently reviewed and analyzed using the Rapid Assessment Process to facilitate key theme and quote identification.

**Results:**

Community partners were predominantly Black, academic researchers and research administrators were predominantly White, and women made up two-thirds of the overall sample. Five key themes were identified: level of partnership equity, partnership collaboration and communication, institutional policies and procedures, level of familiarity with varying fiscal and administrative processes, and financial management expectations. No stakeholders reported best practices for the institutional policies and procedures theme. Cross-cutting challenges included communication gaps between stakeholder groups; lack of or limits in supporting community partners’ fiscal capacity; and lack of collective awareness of each stakeholder group’s processes, procedures, and needs. Cross-cutting best practices centered on shared decision making and early and timely communication between all stakeholder groups in both pre- and post-award processes.

**Conclusions:**

Findings highlight the importance of equitable processes, triangulated communication, transparency, and recognizing and respecting different financial management cultures within community-engaged research. This work can be a springboard used by CTSA institutions to build on available resources that facilitate co-learning and discussions between community partners, academic researchers, and research administrators on fiscal readiness and administrative processes for improved community-engaged research partnerships.

## Abstract 2

Supplemental Digital Content is available in the text.

National recognition of the importance of community and stakeholders’ engagement in clinical and translational research for improving population health and achieving health equity has grown over the past decade. ^1–9^ Community–academic partnerships are essential for high-quality, impactful health research, as such engagement enhances clinical trial design and delivery, increases research relevance and sustainability, improves participant recruitment and retention, and enhances external validity, as well as the public’s receptiveness to research. ^2,10,11^ Federal and nonfederal sponsors (e.g., the National Institutes of Health [NIH], Patient-Centered Outcomes Research Institute, Agency for Healthcare Research and Quality, Institute of Medicine [now the National Academy of Medicine], Centers for Medicare & Medicaid Services) are requiring stakeholder engagement in the design, implementation, and dissemination of research that they fund. ^12–18^

The Clinical and Translational Science Awards (CTSA) Program at the NIH’s National Center for Advancing Translational Sciences places strong emphasis on community engagement and the importance of community–academic partnerships. ^19^ In 2013, the Institute of Medicine reviewed the progress of and made recommendations for the CTSA Program moving forward to improve its efficiency and effectiveness. The resulting CTSA report generated key recommendations on community engagement, including conducting innovative training and mentoring to prepare the next generation of the clinical and translational science workforce and ensuring community engagement across all phases of clinical and translational research. ^16^ The CTSA Program Steering Committee continues to use this report’s recommendations to support the National Center for Advancing Translational Sciences’ strategic plan, which seeks to develop novel processes, share best practices, promote collaboration, and harmonize translational research approaches. ^16,20,21^ One specific objective of the strategic plan is to “engage patients, community members, and nonprofit organizations meaningfully in translational science, and develop and broadly disseminate best practices for patient-focused research.” ^21^ Thus, CTSA institutions have community engagement programs designed to foster collaborative partnerships, enhance public trust in research, and facilitate recruitment and retention of research participants. ^22^

However, efficiently conducting community–academic partnered research remains burdensome. For example, challenges persist in designing and implementing effective community–academic research in which transparent communication and reciprocal relationships foster co-learning and knowledge, skills, and resource sharing. ^23^ Community-engaged research teams face challenges in overcoming academia and community differences, initiating studies when no prior community relationships or trust exists, and balancing competing priorities. ^24^ Inherent in these challenges is ensuring fiscal and administrative (i.e., pre- and post-award grants process) expectations are met by the research team despite labyrinth administrative processes and onerous financial and human resource burdens. Fiscal and administrative processes in community-engaged research require funds to support community involvement, reduced contract negotiation periods with external partners, improved subcontract and subaward execution and monitoring, and communication plans with clear fiscal and administrative expectations. ^23–25^ However, little is known about the influence that academic administrators (i.e., those who manage these processes across the grant cycle) ^26^ have as a key stakeholder group in conducting community-engaged research.

To begin to address this gap, we conducted a qualitative study to identify administrative and fiscal barriers and facilitators to community-engaged research from the perspectives of community, academic, and administrative stakeholders across 4 CTSA institutions. Exploring and comprehending fiscal and administrative bottlenecks from diverse stakeholder perspectives may promote positive proximal (e.g., increased, bidirectional readiness to fiscally collaborate in community-engaged research), intermediate (e.g., more nimble and efficient processes), and distal (e.g., increased trust) outcomes that address the endemic challenges mentioned above.

## Method

### Setting

The North Carolina Translational and Clinical Sciences Institute, the CTSA institution at the University of North Carolina at Chapel Hill (UNC), led this study in collaboration with the CTSA institutions at Medical University of South Carolina, Vanderbilt University Medical Center, and Yale University. The UNC Institutional Review Board approved this study (IRB#15-0849).

### Recruitment and study sample

We used purposive sampling to identify candidate adult (≥ 18 years of age) key informants. Each institution provided candidates’ names and contact information for 3 stakeholder categories:

- Community partners working at a community-based, faith-based, or health-related organization or agency (e.g., local health department, hospital, community health care practice) with any amount of research experience with an academic investigator at a CTSA-affiliated institution;
- Academic researchers working in a CTSA-affiliated institution setting as a research investigator or research staff (e.g., project manager, research associate) with experience working with community partners on at least one research project; or
- Research administrators working in a CTSA-affiliated academic center, departmental business office, or central office for research and grants (e.g., sponsored research office) with experience advising academic researchers who have worked with community partners.

Using the provided list, members of the UNC team (M.E.G., L.C-.E.) sent email invitations to potential participants, followed by email or phone contact (≤ 3 attempts) for nonrespondents. Additional persons were contacted to account for those who declined or did not respond to the invitation, with outreach continuing until 24 participants (2 academic researchers, 2 research administrators, and 2 community partners per CTSA institution) were successfully recruited. Data analysis verified that this was a sufficient sample to achieve thematic saturation. Individuals provided verbal consent to participate in an interview.

### Study design and data collection

Semistructured interviews were conducted in person or via phone and audiorecorded in March–July 2018 by study team personnel at UNC (M.E.G., L.C-.E.). Interviews lasted approximately 40 minutes. Participants were asked about perceived challenges and best practices associated with community-engaged research fiscal and administrative (i.e., the pre- and post-award grants process) processes, as well as desired or relevant resources and trainings (not reported on here due to space constraints; see interview guide in Supplemental Digital Appendix 1 at http://links.lww.com/ACADMED/B55). Academic researchers and research administrators were not offered incentives; however, community partners were offered a $50 gift card for their participation.

Interview audio recordings were transcribed by a member of the UNC team (Adina Black) and a professional transcription company. One UNC team member (M.E.G.) conducted a quality check by reviewing approximately 10% of each transcript for accuracy; if needed, the entire transcript was reviewed.

### Data analysis

Transcribed interviews were analyzed using the Rapid Assessment Process (RAP). ^27^ To facilitate key theme and quote identification, a RAP template was created based on the interview guide. Each transcript was independently reviewed by 2 UNC team members (L.C-.E., M.E.G., A.B., Adina Black, Elisa D. Quarles), who noted key concepts and quotations in a RAP summary sheet. Each pair then met to compare and reconcile responses, creating a final summary sheet. Two analysts (M.E.G., L.C-.E.) reviewed and discussed all final RAP summary sheets to identify common themes and concepts. Emerging themes were identified through discussion and consensus and further refined through the creation of matrices organized by theme and stakeholder group. The terms challenges and best practices were not defined for participants, rather the themes represent compilations of what key informants reported were perceived barriers and facilitators when engaging in fiscal and administrative processes in community-engaged research. To ensure anonymity, demographic data were aggregated across the stakeholder groups.

## Results

The overall study sample was two-thirds female with a community partners sample that was predominantly Black and academic researchers and research administrators samples that were predominantly White (Table 1). The mean number of years participants had spent in their current positions and in conducting, facilitating, and/or supporting community-engaged health research were both over 10 years for each stakeholder group.

**Table 1:**
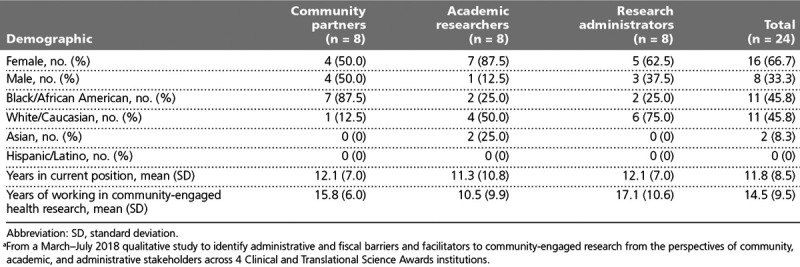
Participant Demographics^a^

Table 2 presents the definitions of the 5 identified key themes, and Appendix 1 provides key examples and sample quotes for the identified themes.

**Table 2:**
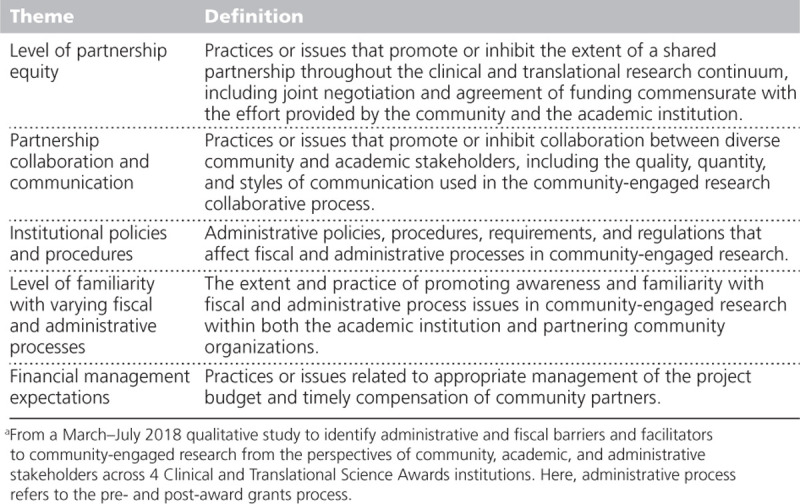
Definitions of the 5 Identified Key Themes^a^

### Perceived challenges by theme

#### Level of partnership equity.

Across all stakeholder groups, participants recognized and identified inequities in research processes, such as high indirect rates, lack of engagement of community partners in the pre- or post-award fiscal decisions, and inequitable opportunities for sustainability beyond the funding period that made it challenging to maintain partnership equity.

… I have the sense at least that it [indirect rate] feels unequal. Especially in the setting of a history of universities often taking from the community and doing research on the community…. Maybe it’s a conversation I don’t like to have because it feels bad. (Academic researcher)

Lack of early inclusion of community partners in the pre-award period, including decisions made without community partners or investigators seeking last-minute feedback on a grant submission, was another challenge to partnership equity noted by participants.

And I didn’t get the whole, completed … grant application until after it had gone in…. I was really alarmed by some of the things that were proposed. I kept saying, “Well, who’s going to do that?” (Community partner)

#### Partnership collaboration and communication.

The challenges to collaboration in the pre- and post-award periods noted by participants included time-related factors (e.g., academic and community partners working at different paces, time needed to build relationships), other existing priorities and competing demands, keeping community partners engaged in the process before the work begins, starting grant administration processes late, missing deadlines, and managing competing schedules at the academic institutions’ fast pace.

… community engagement, it’s a process, it takes time building the relationship, trusting, and lots of times institution folks do not have patience for that. (Community partner)

Participants also indicated that communication challenges exist in the pre- and post-award periods, including email glitches (impacting timely responses), research jargon, varying literacy levels, not receiving project updates in a timely matter, and lack of responsiveness.

If we’re not hearing in a timely fashion back from the community partner, then I’m wondering what their business process is. Are they overwhelmed? Are they just kind of flaky? What’s going on? (Research administrator)

Academic researcher and research administrator stakeholders shared challenges in communicating with community partners about protocol compliance issues or deadlines during the post-award period without damaging the relationship.

It’s like I don’t want to make them upset, but I want them to understand the process. We can’t step out of line. (Academic researcher)

Finally, community partners noted challenges interacting with certain institutional personnel, such as research administrators who were not willing to be contacted directly:

[It] got pretty complicated because the persons who were handling the financial part of the study did not want us to contact them directly…. We had to go through an intermediary…. It also made us feel less valued as a community partner. (Community partner)

#### Institutional policies and procedures.

Confusing, overwhelming, and time-consuming paperwork was a challenge that was recognized by participants, as was a lack of standardized processes.

We’re a community-based nonprofit. I don’t have a business office person who works with NIH grants, who can sit and explain some of the legalese. (Community partner)

Participants noted that staff turnover at academic institutions can lead to a lack of institutional memory or confusion in fiscal-related communications. They described how institutional infrastructure and policies, such as protracted invoicing, budgetary red tape, and burdensome requirements for onboarding community partners to projects (e.g., obtaining institutional affiliate computer credentials and completing human subjects training), can slow progress.

#### Level of familiarity with varying fiscal and administrative processes.

Participants in all 3 stakeholder groups noted challenges in community and academic understanding of fiscal and/or administrative processes. Community partners may lack understanding about academic institutional fiscal processes (e.g., indirect rates) and their grant writing and review process (e.g., the amount of time it takes) or about the rules and regulations of the awards managed by the institution.

It is very important to be very transparent, but sometimes it can be hard when the community partner or organization doesn’t understand hierarchy, the grant, and the processes and how those can work. (Academic researcher)

Likewise, research administrators may lack experience working with community partners and/or an understanding of the compensation processes and needs of community partners.

When we deal with administrative officers or finance folks, most of them don’t really have any experience or training in these partnerships. (Academic researcher)

#### Financial management expectations.

Participants described how community partners are burdened by challenges in navigating institution processes not adapted for community organizations, including having to regularly redo forms and paperwork, being compensated in undesirable ways (e.g., gift cards), facing tax consequences or delays because of paperwork errors, and experiencing payment delays with real consequences for the stakeholder, the partnership, and the project:

As a small nonprofit, sometimes we need that money … it takes 8 weeks for the check to come and we’re already starting working, that’s a long time for someone like us. Usually it requires some sort of invoicing, which inevitably gets sent back. (Community partner)

Participants also noted that some community partners invoiced for unallowable expenses or spent cash advances on unapproved items, leading to owing money, not receiving expected compensation, or compliance issues.

[There have] been times that the partner tried to reallocate money a certain way. And it’s like, “Wait, we can’t do that,” or “You just can’t pay this person extra money because you feel like they deserve more.” (Academic researcher)

Additionally, participants shared that differing financial management procedures between community organizations and academic institutions was a concern.

You get budgets that just have a total, have no detail; that don’t have like broken out personnel, fringe [benefits and insurance]. You have to go back to them multiple, multiple, multiple times to get it to where [the university] will accept it. (Research administrator)

### Perceived best practices by theme

#### Level of partnership equity.

Participants emphasized the importance of community partners’ equitable involvement in the pre-award process, such as understanding their interests, sharing in decision making, involving them in grant writing, paying them for this work, and allowing them to negotiate their role or decline participation in projects that were not feasible for them or that were not aligned with their mission.

I would invite community partners … to be realistic and put a boundary around what you’re able to accomplish. Don’t overpromise. (Research administrator)

They also highlighted the necessity of community partners’ full engagement in the post-award period, including joint budgetary decisions and project governance structures. Additional best practices included community partners serving as fiscal agents for grants and hiring and training community members as research associates.

#### Partnership collaboration and communication.

Participants recommended clear, plain language; early, timely, and regular communication; and clear roles and process information, such as memoranda of understanding, timelines, deadlines, and payment requirements. In addition, they recommended that interactions should be made convenient for community partners (e.g., holding meetings in community spaces), with kickoff meetings when beginning new projects, and the business office being involved throughout the pre- and post-award periods.

As soon as I know […] the community partners involved, I ask our PI [principal investigator] to … have his collaborator put me in touch with my counterpart [in the community organization]. So early on they’re talking science and I’m talking administration. (Research administrator)

#### Institutional policies and procedures.

No stakeholders reported perceived best practices for this theme.

#### Level of familiarity with varying fiscal and administrative processes.

Promoting community partner familiarity with academic institutional policies (e.g., timelines, rules) and processes (e.g., the pre-award process, including potential of not receiving funding) or working with community partners with research experience was noted by participants to be very helpful. To facilitate this, participants discussed offering training and education (e.g., grant writing trainings, pre- and post-award trainings), sharing information about institution fiscal practices or requirements, developing standardized resources (e.g., community partner toolkits), and having community partners review materials and ask questions to ensure understanding.

… review and ask as many questions as can be asked on the front end. Sometimes you don’t always know what to ask until it goes wrong, so then you know for the next time…. (Community partner)

#### Financial management expectations.

Having clearly defined payment structures and financial management procedures were strategies that participants mentioned valuing. For example, participants discussed the importance of documenting and reviewing budgets and spending, adhering to budget justifications and documenting changes, reviewing community paperwork before sending it on to be processed to reduce payment delays, tracking expenditures by the community partners, and having strong financial involvement of principal investigators.

With the community partners and the person responsible for our budget on our end, [we] make sure that all t’s are crossed, all i’s are dotted before we send it off. (Academic researcher)

Participants discussed best practices in paying community partners, noting the importance of cash advances or upfront payments and developing processes to quickly issue payments.

Because as an institution you can wait 30, 60, 90 days maybe, but folks at the community level can’t wait that long…. So, really negotiating quicker turnaround times once invoices are submitted. (Community partner)

## Discussion

To our knowledge, this is the first qualitative study of stakeholders’ perspectives on community-engaged research fiscal and administrative processes, with input from research administrators as well as community partners and academic researchers. Our findings highlight the importance of equitable processes, triangulated communication, transparency, and recognizing and respecting different financial management cultures within community-engaged research; a summary of suggested actions for research teams based on these findings is displayed in Table 3. Further, the differences in demographics between the stakeholder groups in our sample illustrate the need to understand and address racial and gender inequity in community–academic research partnerships, as previously noted by others. ^28,29^ The challenges identified imply that opportunities exist to better align the fiscal processes in community-engaged research with the principles of community engagement. ^22^ For example, institutional fiscal procedures are largely inflexible, requiring community partners to adapt to existing structures without institutional understanding of the community partners’ circumstances and needs. Processes should be established to promote a culture in which academic researchers and research administrators are better prepared to demonstrate authentic team collaboration ^30–32^ and maintain community trust ^33,34^ by releasing equitable control to community partners and by being flexible enough to meet their changing research needs. ^22^ Pursuit of such processes should be a priority across all stakeholder groups and CTSA institutions.

**Table 3:**
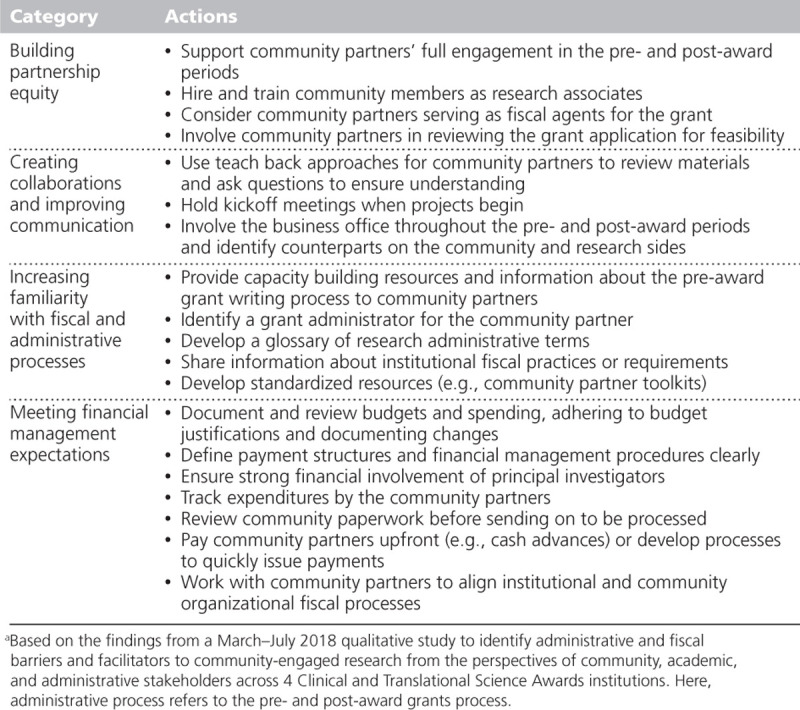
Summary of Suggested Actions for Research Teams^a^

Our findings complement recent national findings that financial management in the pre-award (e.g., budgets, budget justifications) and post-award (e.g., subcontract and subaward monitoring) periods are investigator-identified high-priority areas in vital need of change within academic institutions. ^35,36^ Not only must fiscal processes within academic institutions be improved, but they must also be recognized as key factors in the building and maintenance of partnerships and trust within community-engaged research. ^37,38^

As suggested by our findings, improved communication and transparency among community partners, academic researchers, and research administrators in the pre- and post-award periods are essential. Streamlined, sustainable processes for the setup and financial management of community-engaged research grants are needed and will require buy-in from institutional leadership. Financial relationships can be further optimized by offering capacity building and resources to allow all partners to be engaged in research-related fiscal processes. ^39^ This may include community-led trainings, for example, that can build academic researchers’ capacity to address power dynamics and implement shared leadership in community-engaged research ^40^ and that can provide academic researchers with frameworks for self-assessing institutional capacity to support community-engaged research. ^41^ These efforts should also include research administrators to maximize the benefits to the administrative process. Furthermore, the absence of identified best practices for the theme of institutional policies and procedures (i.e., to reduce institutional bureaucracy) indicates a pressing need for academic institutions and federal funding agencies to commit to improving efficiencies to better support community–academic research partnerships. ^36^

Our results may not be generalizable to all stakeholders conducting community-engaged research through CTSA institutions. However, what we learned in light of proximal, intermediate, and distal outcomes can further guide understanding of and bidirectional communication on efficient financial and administrative policies, processes, and cross-partner needs and foster trust in research settings. Our findings can also inform improved community-engaged research fiscal readiness at CTSA institutions by suggesting places where existing multistakeholder capacity-building work can be enhanced to promote better community–academic research partnerships. ^42–47^ This work can also be a springboard that CTSA institutions can use to build on available resources for collaboratively developing and testing common tools and trainings that facilitate co-learning and triangulated discussions between community partners, academic researchers, and research administrators on fiscal readiness and administrative processes for improved community-engaged research partnerships.

## Conclusions

Optimally, fiscal readiness will help advance community-engaged research by building better community–academic relationships; increasing efficiency; and developing adaptable, scalable suites of best practices, strategies, tools, and trainings that improve the quality of the clinical and translational research enterprise. Our recommendations for future efforts to improve the fiscal and administrative processes in community–academic research are to (1) design best practices and strategies to address communication, regulatory knowledge and streamlining, and capacity building; (2) communicate these best practices and strategies within collaborative academic and community partnerships; and (3) develop tools and trainings that triangulate learning between community partners, academic researchers, and research administrators.

## Acknowledgments:

Adina Black and Elisa D. Quarles served as reviewers in the Rapid Assessment Process. Adina Black provided administrative support in developing this manuscript. Jennifer Teixeira, director of research administration in the Office of Sponsored Research at the University of North Carolina at Chapel Hill, contributed to the conceptualization of this project. The authors thank the stakeholders—community partners, academic researchers, and research administrators—for participating in this study.

## Supplementary Material


