# Supplementary material for: Recognizing Cross-Institutional Fiscal and Administrative Barriers and Facilitators to Conducting Community-Engaged Clinical and Translational Research
Source: Acad Med. 2021 Mar 30;96(4):558–67. doi: 10.1097/ACM.0000000000003893 (PMC7996237; doi:10.1097/ACM.0000000000003893)
Supplement: Supplementary file 1 [file acm-96-558-s001.pdf]

Supplemental digital content for Carter-Edwards L, Grewe ME, Fair AM, et al. Recognizing cross-institutional fiscal and administrative barriers and facilitators to conducting community-engaged clinical and translational research. Acad Med.

Supplemental Digital Appendix 1

Interview Guide

<u>Academic Investigators/Staff:</u>

1) What is one word you would use to describe your experience conducting grant-funded research projects in which community partners receive funding? Why did you choose this word?
2) I'm now going to ask questions about your experience during the **pre-award period** (before grant funding is received). These questions relate to grant funded research projects in which community partners would receive funding.
    a. What has worked well when navigating fiscal & grants management processes during the pre-award period?
        i. [If examples of pre-award period needed: working with community partners on the grant submission process, negotiating MOU with community partner, negotiating indirect funds, determining time necessary to get grant ready to submit, etc.]
        ii. [Alternate phrasing of question 2a] Do you have any best practices to share for navigating fiscal & grants management processes during the pre-award period?
        iii. What would be your definition of a successful fiscal & grants management process during the pre-award period?
            1. What are some indicators or measures you use [or could use] to measure how the pre-award period went?
    b. What challenges or gaps exist for you when navigating fiscal & grants management processes during the pre-award period?
        i. Could you give an example from your experience?
        ii. Why do you think these challenges exist?
        iii. How do you deal with these challenges?
        iv. How do these challenges impact your work?
    c. In the time period after you submit a grant application, but before you hear whether you received funding, in what ways do you engage with your community partners?
3) I'm now going to ask questions about your experience during the **post-award period** (after notice of grant award is received). Again, these questions relate to grant funded research projects in which community partners receive funding.
    a. What has worked well when navigating fiscal & grants management processes during the post-award period?
        i. [If examples of post-award period needed: paying community partners, setting up financial processes, receiving invoices from community partners, etc.]

ii. [Alternate phrasing of 3a] Do you have any best practice to share for navigating fiscal & grants management processes during the post-award period?

iii. What would be your definition of a successful fiscal & grants management process during the post-award period?

1. What are some indicators or measures you use [or could use] to measure how the post-award period went?

b. What challenges or gaps exist for you when navigating fiscal & grants management processes during the post award period?

i. Could you give an example from your experience?

ii. Why do you think these challenges exist?

iii. How do you deal with these challenges?

iv. How do these challenges impact your work?

4) What resources or training would help you or other researchers more effectively engage community partners as paid entities in research?

5) What would be the best way for us to share resources about the fiscal & grants management process with research staff at your institution?

6) Thank you so much for sharing your perspectives today! Before we end, I have a few demographic questions, which we are asking all interview participants:

a. What is your gender?

b. What is your race?

c. Are you Hispanic or Latino?

d. How many years have you worked in your current position?

e. How many years have you worked with community-engaged health research projects?

<u>Business Office Staff or Research Administrators:</u>

1) What is one word you would use to describe your experience being involved in the fiscal and grants management process for research projects in which community partners receive funding? Why did you choose this word?

2) I'm now going to ask questions about your experience during the **pre-award period** (before funding is received). These questions relate to grant funded research projects in which community partners would receive funding.

f. What has worked well when helping investigators & community partners navigate fiscal & grants management processes during the pre-award period?

i. [If examples of pre-award period needed: working with investigators & community partners on the grant submission process, negotiating MOU,

negotiating indirect funds, determining time necessary to get grant ready to submit, etc.]

ii. [Alternate phrasing of 2a] Do you have any best practices to share for helping investigators and community partners navigate fiscal & grants management processes during the pre-award period?

iii. What would be your definition of a successful fiscal & grants administration process during the pre-award period?

1. What are some indicators or measures you use [or could use] to measure how the pre-award period went?

g. What challenges or gaps exist for you when helping investigators & community partners navigate fiscal & grants management processes during the pre-award period?

i. Could you give an example from your experience?

ii. Why do you think these challenges exist?

iii. How do you deal with these challenges?

iv. How do these challenges impact your work?

3) I'm now going to ask questions about your experience during the **post-award period** (after notice of award is received). Again, these questions relate to grant funded research projects in which community partners receive funding.

a. What has worked well when helping investigators & community partners navigate fiscal & grants management processes during the post-award period?

i. [If examples of post-award period needed: paying community partners, setting up financial processes, receiving invoices from community partners, etc.]

ii. [Alternate phrasing of 3a] Do you have any best practices to share for helping investigators and community partners navigate fiscal & grants management processes during the post-award period?

iii. What would be your definition of a successful fiscal & grants management process during the post-award period?

1. What are some indicators or measures you use [or could use] to measure how the post-award period went?

b. What challenges or gaps exist for you when helping investigators & community partners navigate fiscal & grants management processes during the post-award period?

i. Could you give an example from your experience?

ii. Why do you think these challenges exist?

iii. How do you deal with these challenges?

iv. How do these challenges impact your work?

4) [If not addressed previously] What advice would you give to a research team to prepare them for implementing grant-funded research projects in which community partners receive funding?
5) What resources or training would help you or others in similar positions support research projects in which community partners receive financial compensation?
6) What would be the best way for us to share resources about the fiscal & grants management process with staff at your institution?
7) Thank you so much for sharing your perspectives today! Before we end, I have a few demographic questions, which we are asking all interview participants:
    a. What is your gender?
    b. What is your race?
    c. Are you Hispanic or Latino?
    d. How many years have you worked in your current position?
    e. How many years have you worked with community-engaged health research projects?

Community Partners:

1) What is one word you would use to describe your experience working with academic partners on research projects from which you/your organization receive funding? Why did you choose this word?
2) I'm now going to ask questions about your experience during the **pre-award period** (the time period before funding is received, including the time period when you are preparing a research grant submission).
    a. What has worked well when navigating fiscal & grants management processes during the pre-award period?
        i. [If examples of pre-award period needed: working with investigators on the grant submission process, negotiating MOU, negotiating indirect funds, determining time necessary to get grant ready to submit, etc.]
        ii. [Alternate phrasing of question 2a] Do you have any best practices to share for navigating fiscal & grants management processes during the pre-award period?
        iii. What would be your definition of a successful fiscal & grants management process during the pre-award period?
            1. What are some indicators or measures you use [or could use] to measure how the pre-award period went?
    b. What challenges or gaps exist for you when navigating fiscal & grants management processes during the pre-award period?
        i. Could you give an example from your experience?
        ii. Why do think these challenges exist?

iii. How do you deal with these challenges?

iv. How do these challenges impact your work?

c. In the time period after you submit a grant application, but before you hear whether the grant is funded, in what ways do your academic partners engage with you or your organization?

i. How would you *like* your academic partners to engage with you or your organization during this time period?

3) I'm now going to ask questions about your experience during the **post-award period** (the time period after the investigator is notified that they were awarded the grant). Again, these questions relate to grant funded research projects in which community partners receive funding.

a. What has worked well when navigating fiscal & grants management processes during the post-award period?

i. [If examples of post-award period needed: getting paid, setting up financial processes, providing invoices to university, etc.]

ii. [Alternate phrasing of 3a] Do you have any best practices to share for navigating fiscal & grants management processes during the post-award period?

iii. What would be your definition of a successful fiscal & grants management process during the post-award period?

1. What are some indicators or measures you use [or could use] to measure how the post-award period went?

b. What challenges or gaps exist for you when navigating fiscal & grants management processes during the post award period?

i. Could you give an example from your experience?

ii. Why do you think these challenges exist?

iii. How do you deal with these challenges?

iv. How do these challenges impact your work?

4) What resources or training would help your organization better navigate the fiscal & grants management process when partnering with researchers?

5) What would be the best way for us to share resources about the fiscal & grants management process with your organization?

6) Thank you so much for sharing your perspectives today! Before we end, I have a few demographic questions, which we are asking all interview participants:

a. What is your gender?

b. What is your race?

c. Are you Hispanic or Latino?

d. How many years have you worked in your current position?

e. How many years have you worked with community-engaged health research projects?
